# Limitations and Future Aspects of Communication Costs in Federated Learning: A Survey

**DOI:** 10.3390/s23177358

**Published:** 2023-08-23

**Authors:** Muhammad Asad, Saima Shaukat, Dou Hu, Zekun Wang, Ehsan Javanmardi, Jin Nakazato, Manabu Tsukada

**Affiliations:** Graduate School of Information Science and Technology, Department of Creative Informatics, The University of Tokyo, Tokyo 113-8654, Japan; saima@g.ecc.u-tokyo.ac.jp (S.S.); douhu@g.ecc.u-tokyo.ac.jp (D.H.); wangzekun.felix@gmail.com (Z.W.); ejavanmardi@g.ecc.u-tokyo.ac.jp (E.J.); jin-nakazato@g.ecc.u-tokyo.ac.jp (J.N.); mtsukada@g.ecc.u-tokyo.ac.jp (M.T.)

**Keywords:** federated learning, communication efficient, model compression, resource management, client selection, structured updates

## Abstract

This paper explores the potential for communication-efficient federated learning (FL) in modern distributed systems. FL is an emerging distributed machine learning technique that allows for the distributed training of a single machine learning model across multiple geographically distributed clients. This paper surveys the various approaches to communication-efficient FL, including model updates, compression techniques, resource management for the edge and cloud, and client selection. We also review the various optimization techniques associated with communication-efficient FL, such as compression schemes and structured updates. Finally, we highlight the current research challenges and discuss the potential future directions for communication-efficient FL.

## 1. Introduction

**Figure 1:**
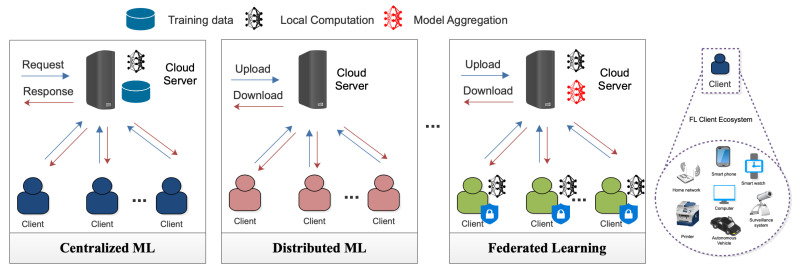
Comparison of FL with conventional centralized machine learning and distributed learning.

Federated learning (FL) is a rapidly growing field that enables multiple clients to train a machine learning model while preserving their data privacy [1]. It has been extensively used in various fields, including healthcare, finance, and social media, where privacy is critical [2,3]. In FL, the clients (devices) perform local training on their respective datasets and then share only the model updates with the server, aggregating the updates to generate a global model [4]. In contrast to conventional centralized machine learning, federated learning (FL) and distributed machine learning offer unique mechanisms for training models across decentralized devices or data sources. However, it is FL that emphasizes collaborative model training with a keen focus on preserving data privacy, minimizing communication overheads, and catering to dynamic and potentially heterogeneous data environments. Distributed machine learning, while reducing computational constraints through parallel processing, often involves more frequent data exchanges without the inherent privacy-preserving design of FL [5]. Figure 1 provides a comprehensive architectural comparison of FL against the traditional centralized and distributed machine learning frameworks. It showcases the nuances in data distribution, model updates, and communication patterns among these paradigms, thus emphasizing the distinct attributes and advantages of FL.

FL is a revolutionary approach in distributed machine learning, promising enhanced data privacy and decentralized model training [6]. However, the communication overheads associated with FL have emerged as a considerable challenge, especially when they pertain to scalability and efficiency. In the FL paradigm, communication costs are bifurcated into two primary categories: upload and download costs. The former encapsulates the data transmitted by clients to the server during the training phase, while the latter accounts for data fetched from the server by the clients [7]. Notably, these costs are influenced by various parameters, such as the dataset size, model intricacy, client count, and network bandwidth [8,9].

The ramifications of these communication costs on FL’s efficiency have been the subject of intensive research. Empirical analyses have ascertained that communication costs can act as substantial constraints, inhibiting the scalability and effectiveness of FL systems [10,11]. To combat these challenges, innovative solutions, such as compression methodologies and model quantization, have been postulated [12,13]. Compression solutions primarily focus on diminishing the magnitude of model updates, thus curtailing upload expenses, whereas model quantization optimizes model parameters’ precision, facilitating further reductions in upload costs.

Moreover, the aspiration for energy-efficient communication systems is a pressing concern that complements the drive for communication efficiency [14]. Reducing communication overheads through FL directly ties into energy savings. Given that every data exchange involves energy consumption, optimizing the FL process impacts the bandwidth and potentially contributes to reduced energy expenditures, a vital consideration for modern communication networks [15,16]. FL’s capacity to minimize data transmission inherently reduces energy consumption, placing it at the forefront of strategies to develop energy-efficient communication networks.

This paper provides an overview of the various communication-efficient FL strategies, including model updating, compression techniques, resource management for the edge and cloud, and client selection. An in-depth look at the different optimization techniques related to communication-efficient FL, such as compression schemes and structured updates, is also included. The potential of communication-efficient FL for emerging distributed applications is discussed, including the benefits and challenges that could arise from its integration into existing distributed systems.

The primary objective of this paper is to analyze the communication efficiency of FL and its impact on system performance. Thus, our following discussion will solely revolve around communication cost, disregarding other aspects of FL. Moreover, to the best of our knowledge, no previous survey has specifically focused on examining the communication cost of FL. Table 1 presents the existing work on FL relevant to our survey.

**Figure 2:**
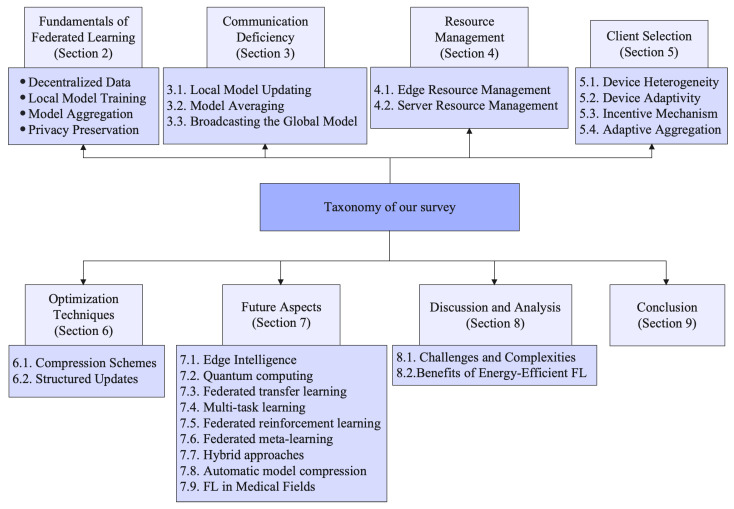
An overview of our survey.

The rest of this paper is organized as follows: Section 2 presents the fundamentals of FL. Section 3 explains the communication deficiency in detail. Section 4 provides the details of resource management strategies in FL. Section 5 presents the importance of client selection in FL. Section 6 presents the optimization techniques of FL. Section 7 presents the potential future directions of FL regarding communication costs. Section 8 provides the discussion and analysis of this survey. Finally, Section 9 concludes this survey paper. A complete overview of this survey is presented in Figure 2.

## 2. Fundamentals of Federated Learning

In this section, we will explore the fundamentals of FL, including the decentralized nature of the data, local model training, model aggregation, and privacy preservation. Understanding these fundamentals is essential for understanding the challenges and opportunities associated with FL and developing approaches to reducing communication costs and improving performance. Table 2 provides a description of the fundamentals of FL, including the advantages and challenges associated with FL. The fundamentals are the following:

- **Decentralized data**: FL involves multiple clients or devices that hold their respective data. As a result, the data are decentralized and not stored in a central location [32,33]. This decentralized nature of data in FL helps preserve the local data’s privacy, but it can also lead to increased communication costs [34]. The decentralized data distribution means more data must be transferred between the clients and the central server during the training process, leading to higher communication costs [35].
- **Local model training**: FL allows each client to perform local model training on its respective data. This local training ensures that the privacy of the local data is preserved, but it can also lead to increased communication costs [36]. The local model updates need to be sent to the central server, which aggregates them to generate a global model. The communication costs of sending these updates to the central server can be significant, particularly when the number of clients or data size is large [37,38].
- **Model aggregation**: After the local model training is completed, the clients send their model updates to the central server for aggregation [39,40]. The server aggregates the model updates to generate a global model, which reflects the characteristics of the data from all the clients [41]. The model aggregation process can lead to significant communication costs, particularly when the size of the model updates is large or the number of clients is high [22,42,43].
- **Privacy preservation**: FL is designed to preserve the privacy of the local data, but it can also lead to increased communication costs [44,45]. The privacy-preserving nature of FL means that the local data remain on the clients, and only the model updates are shared with the central server [46]. However, this also means more data must be transferred between the clients and the server during the training process, leading to higher communication costs.

## 3. Communication Deficiency

**Figure 3:**
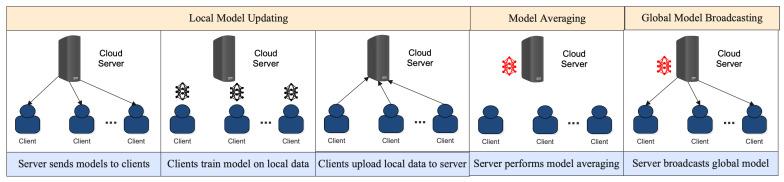
Workflow of communication protocol in FL.

The communication deficiency of FL is an important issue that needs to be addressed for this type of distributed machine learning to be successful. In FL, each client, typically a mobile device, must communicate with a centralized server to send and receive updates to the model [47]. As the number of clients increases, the amount of communication between the server and clients increases exponentially. This can become a major bottleneck, causing the training process to be slow and inefficient. Additionally, communication can be expensive, especially for mobile devices, so minimizing the amount of communication required for FL is important [48]. Figure 3 delves into the intricacies of the FL communication protocol. Beyond merely illustrating the flow, it captures the iterative nature of client–server interactions, highlighting the stages where communication overheads might arise and emphasizing the importance of efficient data exchanges in the FL process. The following section will examine the communication deficiency concerning local model updating and decentralized data training. In Table 3, we highlight the overview of some existing studies on the communication deficiency of FL.

### 3.1. Local Model Updating

Local model updating (LMU) is one of the key techniques used in FL to overcome communication deficiency [65]. In LMU, each participating device trains the shared model on its local data, and only the updated parameters are sent to the central server for aggregation [66,67]. This approach significantly reduces the amount of data that needs to be transmitted over the network, thereby reducing communication costs and latency.

However, several factors can affect the performance of LMU in FL, including the quality and quantity of local data, the frequency of updates, and the selection of participating devices. Below, we discuss some of these factors and their impact on the communication efficiency of LMU in FL:

- **Quality and quantity of local data**: The quality and quantity of local data available on each participating device can significantly impact the performance of LMU in FL. If the local data are noisy or unrepresentative of the global dataset, it can lead to a poor model performance and increased communication costs [68,69]. Moreover, if the quantity of local data is too small, it can lead to overfitting and poor generalization, which can also affect the overall performance of the FL system [52,70]. Several techniques have been proposed to overcome these challenges, such as data filtering and data augmentation [71,72]. Data filtering involves removing noisy or irrelevant data from the local dataset before training the model. In contrast, data augmentation involves generating new data from the existing data to increase the quantity and diversity of the local dataset. These techniques can improve the quality and quantity of local data, thereby improving the performance of LMU in FL.
- **Frequency of updates**: The frequency of updates refers to how often the participating devices send their updated parameters to the central server for aggregation [73,74,75]. A higher frequency of updates can lead to faster convergence and an improved model performance but can also increase communication costs and latency. However, a lower frequency of updates can reduce communication costs but may result in slower convergence and suboptimal model performance. Several approaches have been proposed to balance these trade-offs, such as asynchronous updates and adaptive learning rates [76,77]. Asynchronous updates allow participating devices to update the shared model at their own pace, which can reduce communication cost and latency but may lead to slower convergence. Adaptive learning rates adjust the learning rate based on the frequency of updates, which can improve convergence and reduce communication costs.
- **Selection of participating devices**: The selection of participating devices in FL can significantly impact the performance of LMU [49,78]. If the participating devices are too few or diverse, it can lead to poor model generalization and increased communication costs. Moreover, if the participating devices are biased toward a particular subset of the data, it can lead to a poor model performance and increased communication costs. Several techniques have been proposed to overcome these challenges, such as stratified sampling [79] and weighted aggregation [80]. Stratified sampling involves selecting participating devices based on their similarity to the global dataset, which can improve model generalization and reduce communication costs. Weighted aggregation involves assigning different weights to the participating devices based on their local data quality and quantity, which can improve model performance and reduce communication costs.

### 3.2. Model Averaging

Model averaging is a popular technique used in FL to overcome the communication deficiency problem [81]. In particular, model averaging involves training multiple models on different devices and then combining the models to generate a final model that is more accurate than any individual model [82]. Below, we discuss the model averaging technique in detail and how it can help overcome communication deficiency in FL.

The model averaging technique involves training multiple models using the same training data on different devices. Each device trains its own model using its local data, and the models are then combined to generate a final model that is more accurate than any individual model [83,84]. The models are combined by taking the average of the weights of the individual models. This technique is known as “*Weighted Average Federated Learning*” [85].

Weighted Average FL works as follows. Let $W_{1},W_{2},\ldots ,W_{N}$ be the weights of *N* individual models trained on different devices [86]. The final model is generated by taking the weighted average of the weights of the individual models, where the weights are determined according to their accuracy. That is,

(1) $$\mathrm{Final}\;\mathrm{Weight}=\frac{\left(w_{1}\times W_{1}+w_{2}\times W_{2}+\ldots +w_{N}\times W_{N}\right)}{\left(w_{1}+w_{2}+\ldots +w_{N}\right)},$$

where $w_{1},w_{2},\ldots ,w_{N}$ are the weights determined by the accuracy of individual models, and $W_{1},W_{2},\ldots ,W_{N}$ are the weights of the corresponding models [87].

The weights of the individual models are determined based on their accuracy. Models that perform better on the local data are given higher weights, and models that perform poorly are given lower weights [88]. The weights are updated after each round of training, and the process is repeated until convergence.

The model averaging technique has several advantages over other techniques used in FL. First, it reduces the impact of communication deficiency by allowing each device to train its own model locally. This reduces the amount of communication required between the devices, which is particularly important in scenarios where the communication channel is limited. Second, it improves the accuracy of the final model by combining the knowledge of multiple models. This is particularly useful in scenarios where the local data are diverse and different devices have different data distributions.

In addition, the model averaging technique has been successfully used in several applications, including image classification, natural language processing, and recommendation systems [89]. For example, in image classification, multiple models are trained on different devices using different subsets of the training data [90]. The models are then combined using model averaging to generate a final model that is more accurate than any individual model. This technique has been shown to improve the accuracy of image classification models by up to 20%.

However, there are also some challenges associated with the model averaging technique [91]. One of the main challenges is the selection of the weights of the individual models. The weights should be selected in such a way that they reflect the accuracy of the models. If the weights are not selected correctly, the final model may not be accurate, and the performance may degrade. Another challenge is the convergence of the algorithm. Model averaging requires multiple training rounds, and the algorithm’s convergence can be slow, particularly in scenarios where the local data are diverse [92].

### 3.3. Broadcasting the Global Model

Global model broadcasting is a crucial step in FL, where the locally trained models are aggregated to form a global model [93]. The global model represents the collective knowledge of all the edge devices and is used for making predictions and decisions. The global model must be communicated efficiently and effectively across all devices to achieve a high accuracy and high convergence rate [94]. However, this can be challenging in the presence of communication deficiency. In particular, the central server aggregates the model updates and computes the new global model, which is then broadcasted back to the edge devices [95,96]. There are two main approaches to global model broadcasting in FL: parameter-server-based and peer-to-peer.

In the parameter-server-based approach, a central server acts as a parameter server, which stores and manages the model parameters. The edge devices communicate with the parameter server to upload their local model updates and download the new global model [97]. The parameter server can update the global model by using a synchronous or asynchronous approach. In the synchronous approach, the edge devices upload their local model updates at regular intervals, and the parameter server updates the global model after receiving updates from all devices. In the asynchronous approach, the edge devices upload their local model updates as soon as they are ready, and the parameter server updates the global model in real time.

In the peer-to-peer approach, the edge devices communicate with each other directly to exchange their local model updates [98,99]. The devices can either use a fully connected topology or a decentralized topology to exchange their model updates. In a fully connected topology, each device communicates with all other devices to exchange their local model updates. In a decentralized topology, each device communicates with a subset of other devices to exchange their local model updates [33].

Communication deficiency is a major challenge in global model broadcasting in FL. The deficiency can be caused by a limited bandwidth, high latency, or network congestion [100,101]. The impact of communication deficiency can be severe, leading to slow convergence, a low accuracy, and even a divergence of the global model. In particular, a limited bandwidth can restrict the amount of data that can be transmitted between the edge devices and the central server. This can result in delayed model updates and slower convergence of the global model. High latency can also affect the performance of FL, leading to delayed model updates and the slower convergence of the global model. Network congestion can further exacerbate the problem, as it can cause packet loss and delay in model updates [102].

Several approaches have been proposed to mitigate communication deficiency in global model broadcasting in FL. Compression is one of the most effective approaches, where the model updates are compressed before transmission to reduce the data size [103]. Compression can significantly reduce the amount of data that needs to be transmitted, mitigating the impact of a limited bandwidth and network congestion. Another approach is network optimization techniques that can be used to improve communication efficiency between the edge devices and the central server [104,105]. This can be achieved through various methods, such as adaptive network scheduling, dynamic network reconfiguration, or traffic engineering. These techniques can help optimize the network resources and reduce the impact of network congestion and the latency impact. Model aggregation techniques can also be used to improve the efficiency of global model broadcasting. This can be achieved through various methods, such as federated averaging [106], decentralized optimization [107], or hierarchical aggregation [108]. These techniques can help to reduce the amount of data that needs to be transmitted and improve the convergence rate of the global model.

## 4. Resource Management

**Figure 4:**
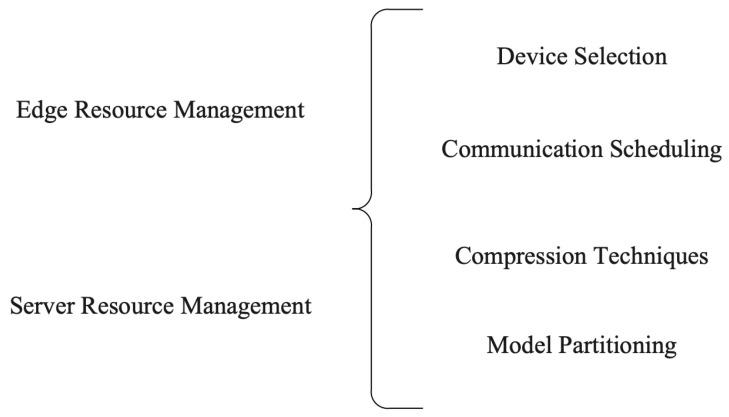
Techniques for clients and server resource management in FL.

Managing resources is critical for the success of FL, which relies on a network of devices to train a machine learning model collaboratively [109]. In addition to computational and communication resources, the availability and quality of edge and server resources can significantly impact the performance of FL systems. In Table 4, we show the categorization of FL resources in terms of the edge and server. In addition, Figure 4 distinctly portrays the myriad techniques deployed for both client and server resource management in the context of federated learning. By effectively managing these resources, we can reduce communication costs and improve the efficiency and accuracy of FL models.

### 4.1. Edge Resource Management

Edge resources refer to the computing and storage resources available on devices participating in the FL process. Edge devices typically have limited resources compared to cloud servers, which makes managing these resources a critical task in FL [110]. Effective edge resource management can help reduce communication costs and improve the overall performance of the FL system.

#### 4.1.1. Device Selection

The first step in edge resource management is selecting appropriate devices for FL. Edge devices include smartphones, tablets, sensors, and other IoT devices. These devices vary in their processing power, memory capacity, battery life, and network connectivity. Therefore, selecting appropriate edge devices is critical for ensuring efficient resource management in FL [111].

One way to select edge devices is based on their processing power. Devices with more processing power can handle more complex machine learning models and computations [64]. However, devices with more processing power also tend to consume more energy, which can limit their battery life. Therefore, selecting devices with the right balance of processing power and energy efficiency is important. Another factor to consider when selecting edge devices is their memory capacity [112]. Devices with more memory can store more data and models, reducing the need for frequent communication with the central server. However, devices with limited memory can bottleneck in FL, especially when dealing with large datasets or models [113].

Network connectivity is another important factor to consider when selecting edge devices. Devices with reliable and high-speed network connectivity can communicate with the central server more efficiently, while devices with poor connectivity may experience delays or errors during communication [114,115]. In general, selecting appropriate edge devices depends on the specific use case and the requirements of the FL system. One common approach is to use a mix of devices with different characteristics to balance the trade-offs between processing power, memory, energy efficiency, and network connectivity.

#### 4.1.2. Communication Scheduling

Communication scheduling is another important aspect of edge resource management in FL. Communication refers to exchanging data and models between edge devices and the central server [62,116]. Communication scheduling involves deciding when and how frequently to communicate and which devices to communicate with.

One strategy for communication scheduling is to schedule communication based on the availability and capacity of the edge devices. Devices with limited resources can be scheduled to communicate less frequently, while devices with more resources can be scheduled to communicate more frequently. This approach can help reduce the overall communication costs of the FL system [117]. Another strategy for communication scheduling is to schedule communication based on the data and model updates [118]. Devices with more recent updates can be scheduled to communicate more frequently, while devices with older updates can be scheduled to communicate less frequently. This approach can help ensure the most relevant and up-to-date data and models are used in the FL process.

In addition, the communication schedule can also consider the network conditions and latency of the edge devices. Devices with poor network conditions or high latency can be scheduled to communicate during periods of low network traffic or when network conditions improve [119]. This approach can help reduce communication errors and delays in the FL system. Effective communication scheduling can help balance the trade-offs between communication costs and model accuracy and ensure the efficient use of edge resources.

#### 4.1.3. Compression Techniques

Compression techniques are important for managing edge resources in FL. In particular, compression techniques involve reducing the data size and exchanging models between edge devices and the central server without sacrificing model accuracy [120].

The need for compression arises due to the limited resources available on edge devices. Edge devices typically have a limited storage capacity and network bandwidth, making transmitting large amounts of data and models challenging [121]. Compression techniques can help reduce the amount of data and models transmitted, making performing FL on edge devices with limited resources possible. There are several techniques for compressing data and models in FL. One common technique is quantization, which involves reducing the precision of the data and models [122]. For example, quantization can be used to represent the data and models as integers with a lower precision instead of transmitting floating-point numbers with a high precision. This can significantly reduce the size of the data and models transmitted without sacrificing much accuracy. Another technique for compressing data and models is pruning, which involves removing redundant or unnecessary parameters from the model [123,124,125]. Pruning can help reduce the model’s size, making it easier to transmit over the network. However, pruning can also lead to a reduction in model accuracy if too many parameters are removed. Another technique for compressing data and models is knowledge distillation, which involves training a smaller model to mimic the behavior of a larger model [96,126]. The smaller model can then be used in place of the larger model, which can help reduce the model’s size without sacrificing much accuracy. Knowledge distillation can be particularly effective when the larger model is complex and has many parameters.

In addition to these techniques, several compression algorithms are specifically designed for FL. For example, federated averaging (FedAvg) is a compression algorithm that involves averaging the model updates from multiple edge devices, which can help reduce the amount of data transmitted between devices [10]. Another algorithm, FedProx, involves adding a penalty term to the loss function to encourage edge devices to stay close to the global model [127]. This can help reduce the amount of data transmitted while maintaining model accuracy. By reducing the size of the data and models transmitted between edge devices and the central server, compression techniques can help reduce communication costs and improve the overall performance of the FL system.

#### 4.1.4. Model Partitioning

Model partitioning is another critical component of FL systems, as it involves dividing the machine learning model into smaller submodels that can be trained on individual devices. Model partitioning aims to reduce the amount of communication required between devices while ensuring that the model’s overall accuracy is not compromised [128].

Several strategies have been developed for model partitioning in FL systems. One common approach is vertical partitioning, where the model is divided based on the features or attributes being used [129]. For example, in an image recognition model, one device may be responsible for training the feature extraction layer, while another device may train the classification layer. This approach can be particularly useful when the model has many features, allowing the devices to focus on a subset of the features [130]. Another approach is horizontal partitioning, where the model is divided based on the data being used [88,131]. For example, each device may train the model on a specific subset of the training data. This approach can be particularly useful when the data are distributed across multiple devices and transferring the entire dataset to a central server would be impractical. A third approach is hybrid partitioning, where a combination of vertical and horizontal partitioning is used to divide the model [132,133]. For example, the model may be partitioned vertically based on the features, and each feature may be further partitioned horizontally across multiple devices. However, the goal should always be to minimize the amount of communication required between devices while maintaining the model’s overall accuracy.

### 4.2. Server Resource Management

Server resource management is a crucial aspect of FL that is responsible for optimizing the utilization of server resources to enhance the efficiency and accuracy of FL models [134,135]. A server’s role in FL is coordinating and managing communication and computation among the participating edge devices. The server needs to allocate computational and communication resources optimally to ensure that the participating devices’ requirements are met while minimizing the communication costs and enhancing the FL model’s accuracy.

#### 4.2.1. Device Selection

Device selection is a critical aspect of server resource management in FL. In an FL system, edge devices train a local model using their data and then communicate the model updates to the server [136,137]. The server aggregates the updates from all devices to create a global model. However, not all devices are suitable for participating in FL for several reasons, such as a low battery life, poor network connectivity, or low computation power. Therefore, the server must select the most suitable devices to participate in FL to optimize resource utilization and enhance model accuracy [138]. The device selection process can be based on several factors: the device computation power, network bandwidth, battery life, and data quality. A popular approach for device selection is to use a machine learning model that predicts the device’s contribution to the global model [139]. The server can use the model’s predictions to select the devices that are likely to provide the most significant contribution to the global model.

#### 4.2.2. Communication Scheduling

The server needs to allocate communication resources optimally to ensure that the participating devices’ updates are timely while minimizing communication costs. In FL, devices communicate with the server over wireless networks, which are prone to communication delays, packet losses, and network congestion [140]. Therefore, the server must effectively schedule communication between devices and the server. The communication schedule can be based on several factors, such as the device availability, network congestion, and data priority. A popular approach for communication scheduling is to use a priority-based scheduling algorithm that prioritizes the communication of high-priority data over low-priority data [141]. The server can use the device’s data priority to schedule the communication effectively, which helps to reduce the communication delay and enhance the model accuracy.

#### 4.2.3. Compression Techniques

In FL, the server receives updates from all participating devices, which can be significant in size. The size of the updates can be reduced by applying compression techniques to the updates before sending them to the server. Compression reduces the communication and the server’s computational costs [142]. The compression techniques can be based on several factors, such as the update’s sparsity, the update’s structure, and the update’s importance.

#### 4.2.4. Model Partitioning

The model partitioning can be based on several factors, such as the model’s size, the model’s complexity, and the available server resources [143]. A popular model partitioning approach is the model distillation technique, which distills the global model into a smaller submodel [144]. The server can use the model distillation technique to partition the model into several submodels that can be trained and stored on different servers [145]. Another approach for model partitioning is to use the model parallelism technique, which splits the model into smaller parts that can be trained simultaneously on different servers [146,147]. The server can use the model parallelism technique to partition the model into smaller submodels that can be trained in parallel, significantly reducing the training time and improving the model accuracy.

## 5. Client Selection

The process of selecting appropriate clients for FL is a critical component of building successful FL systems. In this section, we will discuss various considerations that should be considered when selecting clients for FL, including factors such as device heterogeneity, device adaptability, incentive mechanisms, and adaptive aggregation. In Table 5, we show a comparison of each of those factors.

### 5.1. Device Heterogeneity

Device heterogeneity refers to the variety of devices and their characteristics that participate in an FL system. The heterogeneity of devices presents several challenges in FL, including system heterogeneity, statistical heterogeneity, and non-iid-ness [148].

#### 5.1.1. System Heterogeneity

System heterogeneity refers to differences in the hardware, software, and networking capabilities of the devices participating in the FL system. The heterogeneity of these devices can lead to significant performance disparities and make it difficult to distribute and balance the workload among the devices [149]. These discrepancies can cause communication and synchronization issues, leading to slow convergence rates and increased communication costs. To address these issues, several techniques have been proposed, including device selection algorithms that select devices with similar hardware and software configurations and adaptive communication schemes that adjust the communication frequency and message sizes based on the characteristics of the devices [150,151,152].

#### 5.1.2. Statistical Heterogeneity

Statistical heterogeneity refers to the differences in the data distributions across the devices participating in the FL system. In an ideal FL system, the data should be identically and independently distributed (IID) across all devices, allowing the global model to be trained effectively [153,154]. However, in practice, the data are often non-IID, which can lead to a poor model performance. For example, if one device has significantly more data points for a specific class than others, the global model may become biased toward that class. Several techniques have been proposed to mitigate this issue, including data sampling [155], which involves selecting representative subsets of data from each device to achieve a more balanced distribution across devices, and data aggregation techniques that weigh the contribution of each device’s update based on their local data distribution [156].

#### 5.1.3. Non-IID-Ness

Non-iid-ness refers to the situation where the data distribution across the devices significantly differs from the global distribution. This is a common challenge in FL scenarios, where devices may collect data from different sources or have unique user behavior patterns [157]. The presence of non-iid-ness can lead to slower convergence rates and a poor model performance, as the global model may not accurately represent the data distribution across all devices [21,158]. To address non-iid-ness, several techniques have been proposed, including model personalization, which involves training personalized models for each device based on their local data distribution, and transfer learning, which involves leveraging knowledge learned from similar domains to improve model performance on non-iid data [159,160,161].

### 5.2. Device Adaptivity

Device adaptivity allows devices to adjust their participation in FL, which has emerged as an essential technique to reduce communication costs. Here, we will discuss two critical aspects of device adaptivity: flexible participation and partial updates.

#### 5.2.1. Flexible Participation

Flexible participation allows devices to determine the extent of their involvement in FL based on their capabilities and resources. It allows devices to choose how much data they will contribute, how many communication rounds they will participate in, and when they will participate [162,163]. Flexible participation can significantly reduce communication costs by enabling devices with limited resources to participate in FL without overburdening their systems.

One way to achieve flexible participation is to use dynamic client selection. Dynamic client selection involves selecting clients based on their data quality, availability, and computation capabilities [164]. This approach can significantly reduce communication costs by only selecting a subset of clients to participate in each round of training. Another approach to achieving flexible participation is to use selective transfer learning, where models are selectively transferred from high-capability devices to low-capability devices to minimize communication costs. This approach is particularly effective when training large models with limited resources [165].

#### 5.2.2. Partial Updates

Partial updates allow devices to transmit only a portion of their model updates to the central server instead of transmitting the entire update [166]. This approach can significantly reduce communication costs by reducing the amount of data transmitted between devices. Partial updates can be achieved in several ways, including compressing the model updates, using differential privacy to obscure the update, and using gradient sparsification to reduce the update’s size [167].

Compression techniques, such as quantization, pruning, and sparsification, can be used to reduce the size of the model updates [8]. Quantization involves reducing the precision of the model parameters to reduce their size. Pruning involves removing redundant or insignificant parameters from the model. Sparsification involves setting some parameters to zero to reduce the size of the model update. Differential privacy can be used to obscure the model update by adding random noise to the update [168]. Gradient sparsification can reduce the update’s size by only transmitting the most significant gradient values.

### 5.3. Incentive Mechanism

**Figure 5:**
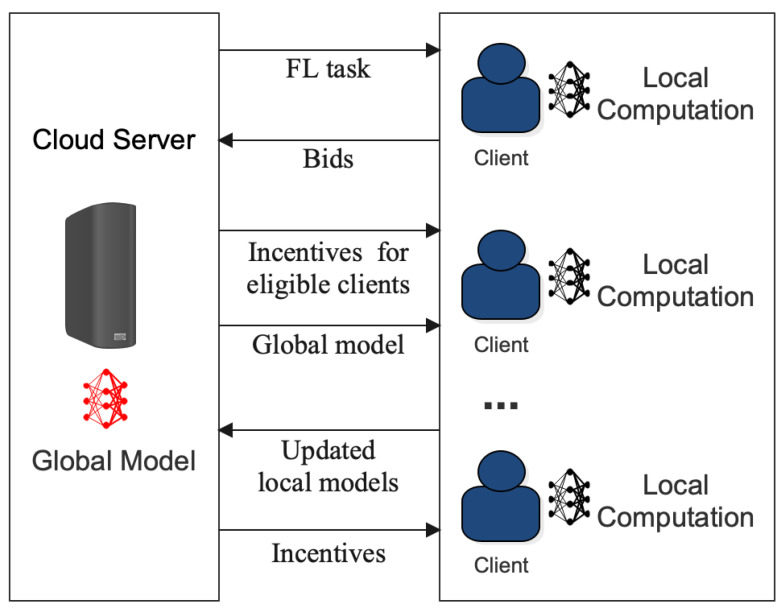
Process of incentive mechanism in FL.

One of the main challenges in minimizing communication costs in FL is incentivizing the clients to cooperate and share their local model updates with the central server. Incentives can encourage clients to participate actively and contribute to the system, leading to a better performance and scalability [97,169,170]. However, designing effective incentive mechanisms is not straightforward and requires careful consideration of various factors. Figure 5 provides a detailed visualization of the FL incentive mechanism. It offers insights into how different stakeholders, from data providers to model trainers, are motivated to participate in the federated ecosystem, ensuring that contributions are recognized and rewarded appropriately, fostering a collaborative and sustainable environment.

Different types of incentive mechanisms can be used to encourage participation in FL. Some of the commonly used incentive mechanisms are explained below:

- Monetary incentives: Monetary incentives involve rewarding the clients with a monetary value for their contributions. This approach can effectively motivate the clients to contribute actively to the system [171]. However, it may not be practical in all situations, as it requires a budget to support the incentive program.
- Reputation-based incentives: Reputation-based incentives are based on the principle of recognition and reputation. The clients who contribute actively and provide high-quality updates to the system can be recognized and rewarded with a higher reputation score [172]. This approach can effectively motivate the clients to contribute to the system actively.
- Token-based incentives: Token-based incentives involve rewarding the clients with tokens that can be used to access additional features or services [173]. This approach can effectively motivate the clients to contribute actively to the system and help build a vibrant ecosystem around the FL system.

The choice of incentive mechanism depends on the system’s specific requirements and the clients’ nature. In general, the incentive mechanism should be designed to align the clients’ interests with the system’s goals. One of the critical factors to consider while designing an incentive mechanism for communication costs in FL is the clients’ privacy concerns [174]. In FL, the clients’ data are typically stored locally on their devices, and only the model updates are shared with the central server. Therefore, the incentive mechanism should not compromise the privacy of the client’s data.

Various privacy-preserving techniques can be used to address the clients’ privacy concerns. For example, differential privacy can be used to ensure that the model updates do not reveal any sensitive information about the client’s data [175]. In this approach, noise is added to the model updates before sharing them with the central server, making extracting any individual information from the updates difficult. Another critical factor to consider while designing an incentive mechanism is the system’s fairness [176]. The incentive mechanism should be designed to ensure that all the clients are treated fairly and that their contributions are appropriately recognized. Fairness can be ensured by designing an incentive mechanism to reward the clients based on their contributions rather than their status or position in the system. Another critical aspect to consider while designing the incentive mechanism is the central server’s level of control over the clients [177]. The incentive mechanism should be designed to ensure that the clients have a certain level of autonomy and control over their data. The clients should be free to decide whether to participate in the system or not, and their contributions should be voluntary.

### 5.4. Adaptive Aggregation

Adaptive aggregation is a method for reducing communication costs in FL systems. In FL, data are typically distributed across multiple devices, and the goal is to train a machine learning model using this decentralized data. To accomplish this, the data are typically aggregated on a central server, which can be computationally expensive and lead to high communication costs [178,179]. Adaptive aggregation seeks to mitigate these costs by dynamically adjusting the amount of aggregated data based on the communication bandwidth of the selected client [180].

The basic idea behind adaptive aggregation is to adjust the amount of data sent to the central server based on the available bandwidth of the devices. This means that devices with slow or limited connectivity can send fewer data, while faster or more reliable connectivity can send more data. Adaptive aggregation can reduce the overall communication costs of FL systems by adapting the amount of data sent [181].

There are several ways that adaptive aggregation can be implemented in FL systems. One approach is to use a threshold-based method, where each device sends a fixed amount of data until its bandwidth is exceeded, at which point it stops sending data [182]. This approach is simple and easy to implement. Still, it may not be very effective at reducing communication costs since it does not consider the variability of communication bandwidth across devices. A more sophisticated approach is a feedback-based method, where the amount of data sent by each device is adjusted based on feedback from the central server [183]. This feedback can be in the form of acknowledgments or error messages, which indicate whether the data received by the server were sufficient to update the model. Devices with faster or more reliable connectivity can send more data, while devices with slower or less reliable connectivity can be limited to sending smaller amounts of data. This approach can be more effective at reducing communication costs since it can adapt to the variability of communication bandwidth across devices. Another approach to adaptive aggregation is to use a learning-based method, where the amount of data sent by each device is adjusted based on past performance [184]. This can be performed using machine learning techniques like reinforcement learning or neural networks. The system can learn to predict the optimal amount of data to send based on the communication bandwidth of the devices and adjust the amount of data sent accordingly. This approach can effectively reduce communication costs since it can adapt to the specific characteristics of the devices in the FL system.

One of the challenges of adaptive aggregation is determining the appropriate amount of data to send for each device. If too few data are sent, the model may not converge to an accurate solution, while if too many data are sent, the communication costs may be excessive [185]. This trade-off can be addressed by using techniques such as cross-validation, which can estimate the model’s performance based on a subset of the data [88]. Another challenge is ensuring that the model is updated in a timely manner despite the variability in communication bandwidth across devices [186]. This can be addressed using techniques such as asynchronous updates, allowing devices to update the model independently and asynchronously [187].

## 6. Optimization Techniques

This section will discuss two key optimization techniques commonly used in FL: compression schemes and structured updates. Table 6 shows the pros and cons of those techniques.

### 6.1. Compression Schemes

Compression schemes involve techniques that reduce the models’ size and gradients exchanged between the client devices and the central server. This is necessary because the communication costs of exchanging large models and gradients can be prohibitively high, especially when client devices have limited bandwidth or computing resources [30,188]. Various compression schemes can be used to address this issue, including quantization, sparsification, and low-rank factorization.

#### 6.1.1. Quantization

Quantization is a popular technique that involves representing the model or gradient values using a smaller number of bits than their original precision [189]. For instance, instead of representing a model parameter using a 32 bit floating-point number, it can be represented using an 8 bit integer. This reduces the number of bits that need to be transmitted and can significantly reduce communication costs. However, quantization also introduces some errors in the model or gradient values, which can affect the quality of the learning process.

#### 6.1.2. Sparsification

Sparsification is another commonly used compression technique that involves setting a large proportion of the model or gradient values to zero [190]. This reduces the number of non-zero values that need to be transmitted, which can result in significant communication savings. Sparsification can be achieved using techniques such as thresholding, random pruning, and structured pruning. However, sparsification can also introduce some errors in the model or gradient values, which can impact the accuracy of the learning process. Some sparsification techniques are described below:

- **Thresholding** is a popular technique for sparsification that involves setting all model or gradient values below a certain threshold to zero [191]. This reduces the number of non-zero values that need to be transmitted, which can result in significant communication savings. The threshold can be set using various techniques, such as absolute thresholding, percentage thresholding, and dynamic thresholding. Absolute thresholding involves setting a fixed threshold for all values, whereas percentage thresholding involves setting a threshold based on the percentage of non-zero values. Dynamic thresholding involves adjusting the threshold based on the distribution of the model or gradient values [192].
- **Random pruning** is another sparsification technique that randomly sets some model or gradient values to zero [123]. This reduces the number of non-zero values that need to be transmitted and can result in significant communication savings. Random pruning can be achieved using techniques like Bernoulli sampling and stochastic rounding [193]. Bernoulli sampling involves setting each value to zero with a certain probability, whereas stochastic rounding involves rounding each value to zero with a certain probability.
- **Structured pruning** is a sparsification technique that sets entire rows, columns, or blocks of the model or gradient matrices to zero [194]. This reduces the number of non-zero values that need to be transmitted and can result in significant communication savings. Structured pruning can be achieved using various techniques like channel, filter, and tensor pruning. Channel pruning involves setting entire channels of the model to zero, whereas filter pruning involves setting entire model filters to zero. Tensor pruning involves setting entire blocks of the model to zero, which can be useful when the model has a structured block-wise pattern. Structured pruning can preserve the underlying structure of the model and can result in higher compression rates than random pruning [195]. Still, it may require more complex implementation and may introduce more errors in the model or gradient values.

#### 6.1.3. Low-Rank Factorization

Low-rank factorization is a compression technique that involves representing the model or gradient matrices using a low-rank approximation [196,197]. This reduces the number of parameters that need to be transmitted and can significantly reduce communication costs. Low-rank factorization can be achieved using techniques such as Singular Value Decomposition (SVD) [198] and Principal Component Analysis (PCA) [199]. However, low-rank factorization can also introduce some errors in the model or gradient values, which can affect the quality of the learning process. The techniques are described below:

- **Singular Value Decomposition (SVD)**: SVD is a matrix factorization technique that decomposes a matrix *X* into three matrices *A*, *B*, and *C* such that $X=ABC^{T}$. Here, *A* and *C* are orthogonal matrices, and *B* is a diagonal matrix containing the singular values of *X*. The script *T* represents the transpose operator, which flips the rows and columns of a matrix. The singular values represent the amount of variation captured by each singular vector. By retaining only the top−k singular values and their corresponding singular vectors, we can approximate the original matrix *X* with a lower rank matrix $X_{k}=A_{k}B_{k}C_{k}^{T}$, where $A_{k}$ and $C_{k}$ are the truncated orthogonal matrices, and $B_{k}$ contains only the top−k singular values [200].
- **Principal Component Analysis (PCA)**: PCA is a dimensionality reduction technique that can be used to compress data. Given a data matrix *X*, PCA aims to find a lower-dimensional representation of *X* that retains the maximum amount of variance. This is achieved by computing the eigenvectors of the covariance matrix of *X* and selecting the top−k eigenvectors corresponding to the largest eigenvalues. The selected eigenvectors form a new orthogonal basis for the data, and the projection of *X* onto this basis yields the lower-dimensional representation of *X* [201].

### 6.2. Structured Updates

Structured updates are another important optimization technique in FL that can reduce communication costs by transmitting only the updates to the changed model parameters. This is necessary because, in many FL scenarios, only a small proportion of the client devices update their local models in each round of communication, and transmitting the entire model can be wasteful [11,202]. Structured updates involve identifying the parts of the model that have been updated and transmitting only those parts to the central server. Various techniques can be used to achieve structured updates, such as gradient sparsification and weight differencing [8].

#### 6.2.1. Gradient Sparsification

Gradient sparsification is a technique used to reduce communication costs in FL. In this technique, only the important gradient values are sent instead of sending the complete gradient information [203]. This can be performed by setting a threshold value and sending only those gradients whose absolute value exceeds the threshold. This threshold can be adjusted depending on the compression and the model’s performance [204]. By reducing the number of gradients sent, the communication costs can be significantly reduced while maintaining the model’s accuracy.

#### 6.2.2. Weight Differencing

Weight differencing is a technique used to reduce communication costs in FL. In this technique, only the differences between the current and previous model parameters are sent instead of sending the entire model parameters [205]. This can be performed by computing the difference between the model parameters at the end of each round and sending only the difference information. This technique reduces the amount of information sent over the network and thus reduces communication costs. However, it requires additional computation at each client to compute the difference and may not be suitable for all scenarios.

## 7. Future Directions

Despite the potential benefits, existing research on FL discusses several challenges associated with communication efficiency. Overcoming those challenges is crucial for harnessing the full potential of FL and realizing its benefits across diverse domains and applications. In Table 7, we briefly summarize the existing research challenges. In addition, below, we explore some possible future directions in FL to reduce communication costs. By leveraging the following techniques, we can improve the efficiency and scalability of FL algorithms and enable the training of machine learning models on increasingly large and diverse datasets.

### 7.1. Edge Intelligence

Edge intelligence is a concept where machine learning models are deployed on the edge devices, such as smartphones, IoT devices, and sensors. By deploying the models on these devices, the communication costs are significantly reduced, as the data does not need to be transmitted to a central server for processing. Instead, the models can be trained locally on the edge devices, and only the model updates need to be communicated to the central server [206,207].

### 7.2. Quantum Computing

Quantum computing has the potential to revolutionize FL by enabling faster and more efficient computations [208]. Quantum computers can perform certain tasks currently infeasible with classical computers, such as factoring large numbers and solving optimization problems. This could lead to significant improvements in the efficiency of FL algorithms, which rely heavily on optimization.

### 7.3. Federated Transfer Learning

Federated transfer learning is a technique where models trained on one device or node can be transferred to another device or node, where they can be fine-tuned on local data. This approach can significantly reduce communication costs, as only the model updates are required to be communicated between the devices rather than the entire model [209].

### 7.4. Multi-Task Learning

Multi-task learning is a technique where a single model is trained on multiple related tasks simultaneously [210]. In FL, this approach can reduce communication costs by allowing the nodes to share their local models, which can be fine-tuned on other related tasks.

### 7.5. Federated Reinforcement Learning

Federated reinforcement learning is a technique where agents learn from their interactions with the environment, and the models are trained in a decentralized manner [211]. This approach can significantly reduce communication costs, as the agents can learn from their local experiences and only communicate the model updates to a central server.

### 7.6. Federated Meta-Learning

Federated meta-learning is a technique where a meta-model is trained on the local models of each node, and the meta-model is used to coordinate the training process [212]. This approach can reduce communication costs by allowing the nodes to share their local models, which can be used to improve the performance of the meta-model.

### 7.7. Hybrid Approaches

Hybrid approaches combine multiple techniques to achieve a better performance and reduce communication costs [213]. For example, a hybrid approach could combine edge intelligence with federated transfer learning, where models are trained on edge devices and transferred to a central server for fine-tuning.

### 7.8. Automatic Model Compression

Automatic model compression is a technique where machine learning models are compressed to reduce their size and complexity, which can significantly reduce communication costs [214]. This technique can be used with other approaches, such as federated transfer learning, to further reduce communication costs.

### 7.9. Federated Learning in Medical Fields

As federated learning (FL) continues its integration with the burgeoning realm of the Internet of Medical Things (IoMT), buttressed by the advanced capabilities of 6G, new horizons in healthcare appear imminent. The study [215] offers a glimpse into this future, showcasing 6G-enhanced FL in IoMT. Emerging challenges and opportunities in this confluence include enhancing real-time health monitoring and diagnostics while ensuring robust data privacy. The next frontier likely involves crafting tailored communication-efficient techniques that can accommodate the unique demands of medical diagnosis and treatment. There is a palpable anticipation for a healthcare paradigm where FL and 6G seamlessly intertwine, catalyzing more personalized, timely, and secure patient care [216]. Future endeavors in this domain will undoubtedly focus on harnessing these synergies for optimal healthcare outcomes.

## 8. Discussion and Analysis

While this survey has comprehensively detailed techniques addressing communication efficiency in FL, it is paramount to understand their inherent challenges, complexities, and potential benefits.

### 8.1. Challenges and Complexities

The juxtaposition of local model updating, model averaging, and broadcasting the global model hints at a delicate balance: optimizing one aspect can inadvertently impact another, leading to unforeseen communication bottlenecks.

Resource management, especially on the edge versus server-side, is not a straightforward binary. Factors like unpredictable client availability, diverse resource capabilities, and fluctuating network conditions make universal solutions elusive. The prominence given to client selection is noteworthy; yet, the task is not trivial. Deciding on “the most appropriate devices” involves not just current resource metrics but predictive insights into their future states, adding another layer of complexity.

Moreover, while optimization techniques like compression schemes and structured updates promise reduced communication costs, they come with their caveats. For instance, aggressive model compression might reduce data transfer but could also lead to degraded model accuracy. Structured updates, although efficient, may not always align with the non-i.i.d data distributions often seen in FL setups.

In light of the future directions presented, it is clear that while advancements such as quantum computing and federated meta-learning offer exciting prospects their practical application in FL will introduce new challenges. It is imperative that, as we move forward, we are not just devising solutions but anticipating the trade-offs they bring to the fore.

### 8.2. Benefits of Energy-Efficient FL

Energy efficiency has recently emerged as an indispensable pillar in the realm of communication systems, predominantly due to the burgeoning demand for connected devices and the skyrocketing data exchange volumes. FL, with its inherent design, lends itself beneficially to this scenario. Some of its benefits are listed below:

- Reduced data transmission: At its core, FL minimizes the need for data centralization. Instead of transmitting extensive datasets, devices share compressed model updates. This direct reduction in data transmission not only conserves bandwidth but also considerably reduces the energy expended in the communication process, given that data transmission and reception are among the most energy-intensive operations in wireless communication.
- Decentralized computation: In FL, computations are performed at the edge, on user devices themselves. This decentralization aids in leveraging the collective computational prowess of these devices, reducing the burden on centralized servers. Consequently, servers consume less energy for computations, ensuring a more balanced and energy-efficient system.
- Intelligent client participation: Energy efficiency in FL is not just about reducing communication. It extends to judiciously determining which clients participate in the training. By selecting devices that are currently charging or have high battery levels, FL processes can minimize battery drain issues, leading to a more sustainable execution of federated tasks.
- Adaptive communication protocols: Modern FL implementations have started employing adaptive communication techniques. By assessing the network’s current state, these techniques modulate the frequency and size of model updates. Such dynamism ensures that devices communicate optimally, preserving energy in low-bandwidth or unstable network conditions.
- Synergy with modern hardware: With the advent of energy-efficient hardware tailored for AI and ML tasks, FL can further amplify energy savings. By integrating with low-power neural network accelerators, for instance, the computational aspect of FL becomes even more energy efficient.

While energy efficiency introduces undeniable advantages, it is paramount to integrate it thoughtfully into the FL paradigm. The challenge is ensuring that the pursuit of energy savings does not compromise the model’s accuracy or the system’s responsiveness.

## 9. Conclusions

This survey paper has thoroughly analyzed the limitations and future aspects of communication costs in federated learning. We have explored the fundamentals of federated learning, the challenges associated with communication deficiency, resource management, client selection, and optimization techniques. The survey has highlighted the need to address communication costs to improve the efficiency and scalability of federated learning. The future directions of federated learning with respect to communication costs have also been identified. This survey paper provides a valuable resource for researchers and practitioners working on federated learning and inspires further research in this area.

## Figures and Tables

**Table 1:** Existing surveys and their primary focus.

Reference	Year	Focus	Communication Constraints	Challenges
[1]	2021	Characteristics and the current practical application of FL	Yes	Network heterogeneity
[17]	2023	Threats and vulnerabilities of FL	No	Adversarial attacks
[18]	2021	Categorization of FL	Partially discussed	Design factors
[3]	2020	Comparison of different ML deployment architectures and in-depth investigation on FL	Partially discussed	Architectural robustness
[19]	2021	Advances and open challenges of FL	No	Privacy and communication
[20]	2021	Characteristics of edge FL	Yes	Security and privacy
[21]	2021	Non-identical and non-independent data distribution in FL	Partially	Communication efficiency
[22]	2022	FL in smart healthcare	No	Design factors
[23]	2023	Blockchain empowered FL	No	Privacy and security
[24]	2022	Security aspects of FL	No	Privacy and security
[25]	2022	Implementation of FL in centralized, decentralized, and heterogeneous approach	Partially discussed	Network heterogeneity
[26]	2022	Integration of FL with industrial IoT	No	Privacy preservation
[27]	2023	FL in wireless networks	Yes	High communication costs
[28]	2023	Review of existing studies on communication constraints in FL	Yes	Communication costs
[29]	2023	Threats to and flaws in the FL strategy	No	Privacy and Security
[30,31]	2020	FL in mobile edge computing	Partially discussed	Design factors
[32]	2020	Personalization of FL	No	Client selection

**Table 2:** Fundamentals of FL.

Category	Description
Definition	FL is a machine learning setting where the goal is to train a model across multiple decentralized edge devices or servers holding local data samples, without explicitly exchanging data samples.
Key Components	The main elements of FL include the client devices holding local data, the central server that coordinates the learning process, and the machine learning models being trained.
Workflow	The typical FL cycle is as follows: (1) The server initializes the model and sends it to the clients; (2) Each client trains the model locally using its data; (3) The clients send their locally updated models or gradients to the server; (4) The server aggregates the received models (typically by averaging); (5) Steps 2–4 are repeated until convergence.
Advantages	The benefits of FL include (1) privacy preservation, as raw data remain on the client; (2) a reduction in bandwidth usage, as only model updates are transferred, not the data; (3) the potential for personalized models, as models can learn from local data patterns.
Challenges	FL faces several challenges, including (1) communication efficiency; (2) heterogeneity in terms of computation and data distribution across clients; (3) statistical challenges due to non-iid data; (4) privacy and security concerns.
Communication Efficiency Techniques	Communication efficiency can be improved using techniques, such as (1) federated averaging, which reduces the number of communication rounds; (2) model compression techniques, which reduce the size of model updates; (3) the use of parameter quantization or sparsification.
Data Distribution	In FL, data are typically distributed in a non-iid manner across clients due to the nature of edge devices. This unique distribution can lead to statistical challenges and influence the final model’s performance.
Evaluation Metrics	Evaluation of FL models considers several metrics: (1) global accuracy, measuring how well the model performs on the entire data distribution; (2) local accuracy, measuring performance on individual client’s data; (3) communication rounds, indicating the number of training iterations; (4) data efficiency, which considers the amount of data needed to reach a certain level of accuracy.

**Table 3:** Existing research focusing on communication deficiency in FL.

Reference	Focus	Overview
[49]	Client selection	The algorithm recognizes the non-IID degrees of clients and chooses those with lower degrees of non-IID data to train the models with higher frequency.
[50]	Client selection	Optimizes the trade-off between maximizing the number of selected clients and minimizes the energy drawn from batteries for the selected clients in FL.
[51]	Resource management	The study uses cluster heads to communicate with the cloud server through edge aggregation, where clients upload their local models to their respective cluster heads. A joint communication and computation resource management scheme is also formulated through efficient client selection to achieve global cost minimization.
[52]	Client selection	The study divides clients into tiers based on their training performance. It selects clients from the same tier in each training round to mitigate the straggler problem. It employs an adaptive tier selection approach to update the tiering on the fly based on the observed training performance and accuracy.
[53]	Communication efficiency	The paper proposes the "In-Edge AI" framework that integrates deep reinforcement learning and FL with mobile edge systems in order to optimize mobile edge computing, caching, and communication.
[54]	Edge resource management	The study proposes a DTWN model and designs an edge association problem armed with FL. A multi-agent deep reinforcement learning-based algorithm is proposed to solve the problem. In addition, the study considers an edge association and communication resource allocation problem to minimize communication costs.
[55]	Edge resource management	The paper proposes a framework called concurrent federated reinforcement learning. Specifically, it protects the privacy of both the server and the edge node with the assistance of blockchain.
[56]	Edge resource management	The paper proposed an FL framework, which can securely update the data with the help of parallel blockchains. It considers a two-phase commit protocol and defines an auction scheme based on ML for price optimization.
[57]	Incentive mechanism	The paper considers a framework of a privacy-preserving incentive mechanism for encouraging the users to join the network. Specifically, the paper makes an extremely rigorous convergence analysis and derives a set of optimal contracts under the constraints of security demands and budget costs for each worker in the scenario.
[58]	Structured updates	The study shows an FL framework for autonomous driving. With the help of MEC nodes and blockchain, the system can achieve a lower latency and more accurate results between the vehicles, even if there are malicious vehicles and MEC nodes.
[59]	Incentive mechanism	The paper proposes an FL-based autonomous vehicle controller. To explain it deeper, the study uses a contract-theoretic incentive mechanism to speed up the process. It considers optimization methods to decrease the communication and computation cost for the system.
[60]	Incentive mechanism	The paper proposes a coded FL method that is based on an evolutionary game and a deep learning method to allocate the resource intelligently. The results show that the study mitigates the overall system computation and communication latency.
[61]	Optimization technique	The paper designs a client–edge–cloud hierarchical FL architecture. It develops an HierFAVG algorithm to allow edge servers to aggregate models partially to gain a higher efficiency.
[62]	Client selection	The study proposes a two-level hierarchical FL framework and designs two incentive mechanisms for resource allocation. The cluster selection mechanism of workers is based on an evolutionary game, and one deep-learning-based auction mechanism is designed for the model owner’s selection of cluster heads.
[63]	Resource management	The paper considers a maximum model accuracy problem of the wireless FL under the limited training time and latency constraint. It proposed a joint device scheduling and resource allocation policy.
[64]	Client selection	The study presents a Clients’ Eligibility Protocol (CEP) to work with heterogeneous clients in practical industrial scenarios efficiently. The CEP uses a trusted authority to calculate the client’s eligibility score based on local computing resources, such as the bandwidth, memory, and battery life, and selects the resourceful clients for training.

**Table 4:** Categorization of FL resources.

Resource	Edge Resource	Server Resource
Data Storage	Local Storage	Distributed Storage
Data Aggregation	Local Aggregation	Distributed Aggregation
Data Processing	Local Processing	Cloud Processing
Data Security	Local Encryption	Cloud Encryption

**Table 5:** Comparison of factors that can be considered for client selection in FL.

Device Heterogeneity	Device Adaptability	Incentive Mechanism	Adaptive Aggregation
Categorize devices	Assess device capability	Assign rewards	Aggregate according to device type
Evaluate device resources	Monitor device performance	Balance rewards	Adjust aggregation strategy
Consider device availability	Check device compatibility	Set rewards based on participation	Consider data privacy
Analyze device specifications	Identify device limitations	Assign rewards based on data quality	Adapt to changes in data distribution
Evaluate device trustworthiness	Assess device reliability	Offer rewards for data computation	Change aggregation frequency
Consider device latency	Determine device storage capacity	Provide rewards for data transmission	Monitor device performance
Check device battery level	Examine device memory usage	Create rewards for data accuracy	Adapt to changing device configurations

**Table 6:** Pros and cons of optimization techniques in FL.

Technique	Pros	Cons
**Compression Schemes**		
Quantization	Reduced communication	Information loss
Sparsification	Lower bandwidth usage	Increased computation
Low-rank factorization	Efficient storage	Complexity in updating
**Structured Updates**		
Gradient sparsification	Reduced communication	Limited expressiveness
Weight differencing	Low memory requirement	Sensitivity to noise

**Table 7:** Summary of existing research challenges in FL related to communication efficiency.

Research Challenge	Brief Description
High Communication Overhead	FL requires transferring large amounts of data, which can lead to high communication costs.
Data Heterogeneity	Differences in data distribution across devices can affect model performance and require efficient communication strategies.
Latency	Variations in network conditions and device capabilities can cause latency issues, requiring efficient communication solutions.
Bandwidth Limitations	A limited bandwidth can cause slow model training and update propagation. The efficient use of the available bandwidth is a challenge.
Stragglers	Some devices may be slow to compute updates or fail to send updates, slowing down the learning process. The efficient handling of stragglers can improve communication efficiency.
Scalability	As the number of participating devices increases, efficiently managing communications becomes more challenging.
Security	Efficiently ensuring secure and privacy-preserving communication is a significant challenge.
Device Failures	Devices may fail or drop out during the learning process, requiring robust communication protocols to handle these situations.
Resource Constraints	Devices participating in FL may have different computational resources, which can create challenges for efficient communication.
Data Synchronization	Ensuring all devices have the latest model updates for efficient learning can be a challenge, especially given the asynchronous nature of FL.
Noise in Gradients	Due to the decentralized nature of FL, there can be a high level of noise in the gradient updates, affecting the overall communication efficiency.
Compressed Communication	Due to bandwidth limitations, it may be necessary to compress data during transmission, which can lead to a loss of information and affect the learning process.

## Data Availability

The original contributions presented in the study and included in the article; further inquiries can be directed to the corresponding author.
